# Case-Mortality in Pneumonia

**Published:** 1900-02-24

**Authors:** 


### CASE-MORTALITY IN PNEUMONIA.

The extraordinary differences which may be shown by the statistics of different hospitals in regard to so well-marked a disease as pneumonia was well illustrated by the remarks made at a recent meeting of the Liverpool Medical Institution, when Dr. Nathan Raw read a paper upon the pathology and treatment of this disease. He based his observations upon 1,074 cases which had occurred during a period of 12 years. The general death-rate of the whole series of cases was, he said, 23·4 per cent. During the past two and a half years, however, 317 cases of pneumonia had been admitted into Mill Road Infirmary (including all forms of pneumonia and those dying soon after admission), with a total death-rate of 34 per cent. Dr. Waters, however, speaking of the statistics of other hospitals, showed how largely the mortality varied according as complicated cases were included or not, and he tabulated all the cases occurring in his hospital practice, to the number of 151, showing that the mortality among them was 7·28 per cent. Probably Dr. Carter hit the right key in saying that on clinical grounds one must recognise two different forms of pneumonia, the one simple and tending to recovery, the other septic and very dangerous, although he did not define what he meant by "septic." If we are to take it that "septic" cases are really cases of mixed infection—cases in which the condition set up by the development of the pneumococcus has given an opportunity to other septic micro-organisms, then it is not difficult to understand that pneumonia in a workhouse infirmary, receiving the sweepings of the slums, might well have a higher case-mortality than is met with in a hospital receiving cases much more representative of the average type. Age, however, has much to do with mortality from this disease. Dr. Wilson Fox collected 15,485 cases from various authors, the whole series giving a mortality rate of 19·3 per cent. If, however, the cases under 20 years of age were separated from the rest they were found to have a mortality rate of only 7·7 per cent., against one of 22·6 per cent. for those over 20 years of age. Dr. Hector Mackenzie[1] again has lately collected the cases of pneumonia which have occurred at St. Thomas's Hospital during a period of 19 years, and he finds that there were 273 deaths out of 1,392 cases, giving a mortality of 19·6 for all ages. But, on sorting out those under 20 years of age, it appears that the mortality in these cases was but 5 per cent., while among those over 20 there was a mortality of 31·4 per cent. These figures not only give us an idea of the wide range in the fatality of this disease, but show how important it is to consider the question of age in discussing the bearing of the fatality in any given series of cases.

[1] Practitioner, January.

